# The type IIS restriction enzyme MmeI can cut across a double-strand break

**DOI:** 10.1007/s11033-023-08375-8

**Published:** 2023-04-08

**Authors:** Maliha Tasnim, T. Jacob Selph, Jason Olcott, Jonathon T. Hill

**Affiliations:** Department of Cell Biology and Physiology, Brigham Young University, Provo, UT 4005, 84602 USA

**Keywords:** MmeI, Type IIS restriction enzymes, Restriction modification, Restriction digestion, DNA digestion

## Abstract

**Background:**

Type-IIS restriction enzymes cut outside their recognition sites, allowing them to remove their binding sites upon digestion. This feature has resulted in their wide application in molecular biology techniques, including seamless cloning methods, enzymatic CRISPR library generation, and others. We studied the ability of the Type-IIS restriction enzyme MmeI, which recognizes an asymmetric sequence TCCRAC and cuts 20 bp downstream, to cut across a double-strand break (DSB).

**Methods and results:**

We used synthetic double-stranded oligos with MmeI recognition sites close to 5′ end and different overhang lengths to measure digestion after different periods of time and at different temperatures. We found that the MmeI binding and cutting sites can be situated on opposite sides of a DSB if the edges of the DNA molecules are held together by transient base-pairing interactions between compatible overhangs.

**Conclusion:**

We found that MmeI can cut across a DSB, and the efficiency of the cutting depends on both overhang length and temperature.

## Introduction

Prokaryotic type IIS restriction-modification systems have provided essential tools for in vitro DNA manipulation and have enabled several prominent molecular techniques, including golden gate and other seamless cloning methods [1, 2], MetClo [3], enzymatic CRISPR library generation [4, 5], site-directed mutagenesis [6, 7], and others. These enzymes are notable because they recognize asymmetric sequences and cut outside their binding site, allowing them to remove their own binding site upon digestion [8]. In addition, many of these enzymes modify their binding site to prevent further recognition at the same location. For example, the type IIS enzyme MmeI recognizes the non-palindromic sequence TCCRAC and cuts 20 bp downstream while subsequently methylating the A in its recognition sequence [9–13]. It is also a type IIL enzyme as it only modifies a single strand for host protection [12]. Previous research on MmeI has shown that it requires two or more sites for efficient cleavage. These sites can be on the same DNA, or the second site can be provided on a second oligo in trans [14]. The relative orientation of recognition sites does not matter, as DNA looping can lead to dimerization for any combination of orientations [14]. When the MmeI recognition site is located at the edge of the oligo and oriented such that the reach of the endonuclease domain extends beyond the end of the oligo, MmeI can bind and participate in making cleavage complexes but will not actively cut [12, 14]. However, it is unclear what will happen if there is a double-stranded break (DSB) between the DNA binding domain and the endonuclease domain. Here, we show that MmeI can cut across a DSB if a complementary overhang is present in both DNA substrates. This cutting is as efficient or more efficient as cutting within the same oligo and does not require MmeI recognition sequences on both sides of the break. We propose that MmeI can bind to its recognition sequence and cut rapidly when a complementary overhang in a second DNA transiently base pairs with the MmeI site containing DNA.

## Materials and methods

### Digestion

MmeI (SAM included), rCutSmart™ buffer, and DNA ladders were ordered from New England Biolabs. Single-stranded (SS) synthetic oligos were acquired from Integrated DNA Technologies (Table 1). Double-stranded oligos with overhangs of different sizes were synthesized by annealing the SS oligos. Each DNA molecule type was synthesized at distinct lengths to allow them to be clearly identified on a gel. The shorter DNA molecule (118 bps) contained a MmeI site with varying lengths of overhang (G, GG, CGG), whereas the longer one (164 bps) did not have any MmeI site and had compatible overhangs (C, CC, CCG). To analyze the effect of overhangs, digestion was done by incubating 130 ng of the shorter DNA substrate (1.7 pmol) and 181 ng of the longer DNA molecule (1.7 pmol) with 4 units (1.28 pmol) of MmeI in rCutsmart buffer (20mM Tris-Albumin, pH 7.9) in a 20 ul reaction. For the DNA (283 bps) containing 1 MmeI site without any DSB, 312 ng of the substrate was used in a 20 ul reaction to ensure an identical molar ratio. To stop each reaction at designated time points, 15 ul of stop solution (50 mM EDTA, pH 8.0, 50% glycerol, 0.05% bromophenol blue) was added to the digestion reaction.

**Table 1:** DNA Oligos used for digestion experiments. Oligos were ordered and hybridized in pairs as indicated. MmeI sites are shown in bold, and overhangs are underlined

Top strand for construct with MmeI recognition sequence (bold)	CTGTAATAAGAGATGATCGTTAGCAGGTGGTCCATTTCGTGCTCGCTATCAGTGCATGTTACATTGATCCTCATACATGCGTAATGATGGAAGTATGCATTGTCAGGATGGTTCCGAC
Bottom strand for construct with MmeI recognition sequence (bold) with G overhang (underlined)	G**GTCGGA**ACCATCCTGACAATGCATACTTCCATCATTACGCATGTATGAGGATCAATGTAACATGCACTGATAGCGAGCACGAAATGGACCACCTGCTAACGATCATCTCTTATTACAG
Bottom strand for construct with MmeI recognition sequence (bold) with GG overhang (underlined)	GG**GTCGGA**ACCATCCTGACAATGCATACTTCCATCATTACGCATGTATGAGGATCAATGTAACATGCACTGATAGCGAGCACGAAATGGACCACCTGCTAACGATCATCTCTTATTACAG
Bottom strand for construct with MmeI recognition sequence (bold) with CGG overhang (underlined)	CGG**GTCGGA**ACCATCCTGACAATGCATACTTCCATCATTACGCATGTATGAGGATCAATGTAACATGCACTGATAGCGAGCACGAAATGGACCACCTGCTAACGATCATCTCTTATTACAG
Top strand for construct with no recognition sequence	TACCCTACACTCTCTCTTTACCAAGCGTGCCTGAAGGACTGAAGACCATCTGGAAGTACCGTTTCTAGTAGCATACATTATACGAAGTCAGGATTGTTAGTTACGTCATACATCAAAGTCAAGCTCAACACGTGCATCAAGAATATTATTCATACTGACGTTG
Bottom strand for construct with no MmeI recognition sequence with C overhang (underlined)	CCAACGTCAGTATGAATAATATTCTTGATGCACGTGTTGAGCTTGACTTTGATGTATGACGTAACTAACAATCCTGACTTCGTATAATGTATGCTACTAGAAACGGTACTTCCAGATGGTCTTCAGTCCTTCAGGCACGCTTGGTAAAGAGAGAGTGTAGGGTA
Bottom strand for construct with no MmeI recognition sequence with CC overhang (underlined)	CCCAACGTCAGTATGAATAATATTCTTGATGCACGTGTTGAGCTTGACTTTGATGTATGACGTAACTAACAATCCTGACTTCGTATAATGTATGCTACTAGAAACGGTACTTCCAGATGGTCTTCAGTCCTTCAGGCACGCTTGGTAAAGAGAGAGTGTAGGGTA
Bottom strand for construct with no MmeI recognition sequence with CCG overhang (underlined)	CCGCAACGTCAGTATGAATAATATTCTTGATGCACGTGTTGAGCTTGACTTTGATGTATGACGTAACTAACAATCCTGACTTCGTATAATGTATGCTACTAGAAACGGTACTTCCAGATGGTCTTCAGTCCTTCAGGCACGCTTGGTAAAGAGAGAGTGTAGGGTA
Top strand of double strand construct with MmeI recognition sequence (bold) configured to cut within the same molecule	CTGTAATAAGAGATGATCGTTAGCAGGTGGTCCATTTCGTGCTCGCTATCAGTGCATGTTACATTGATCCTCATACATGCGTAATGATGGAAGTATGCATTGTCAGGATGGT**TCCGAC**CCCAACGTCAGTATGAATAATATTCTTGATGCACGTGTTGAGCTTGACTTTGATGTATGACGTAACTAACAATCCTGACTTCGTATAATGTATGCTACTAGAAACGGTACTTCCAGATGGTCTTCAGTCCTTCAGGCACGCTTGGTAAAGAGAGAGTGTAGGGTA
Bottom strand of double strand construct with MmeI recognition sequence (bold) configured to cut within the same molecule	TACCCTACACTCTCTCTTTACCAAGCGTGCCTGAAGGACTGAAGACCATCTGGAAGTACCGTTTCTAGTAGCATACATTATACGAAGTCAGGATTGTTAGTTACGTCATACATCAAAGTCAAGCTCAACACGTGCATCAAGAATATTATTCATACTGACGTTGGG**GTCGGA**ACCATCCTGACAATGCATACTTCCATCATTACGCATGTATGAGGATCAATGTAACATGCACTGATAGCGAGCACGAAATGGACCACCTGCTAACGATCATCTCTTATTACAG

### Gel Electrophoresis

Each in vitro digestion was analyzed by agarose gel electrophoresis with a 4% MetaPhor™ agarose with 1x TAE running buffer and DNA size standards at 90 V for 1 h and 20 min. Images were visualized using an Alpha Innotec gel viewer, and subsequent densitometry analysis was done with ImageJ.

### Statistical analysis

**Fig. 1:**
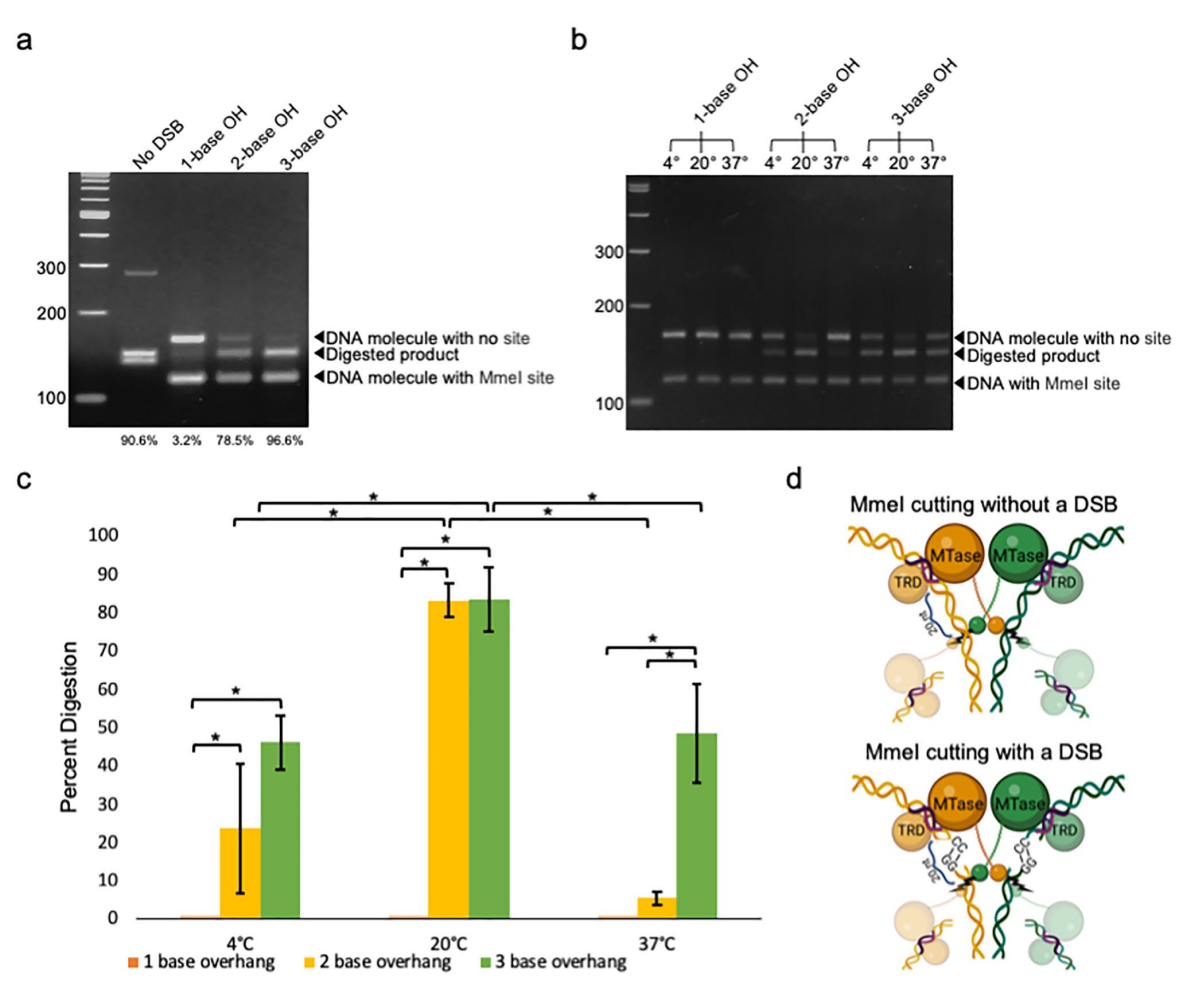
Comparison of cutting rates in the presence or absence of a DSB. (a) Gel showing a comparison of the amount of digestion among single DNA with one MmeI recognition site and similar sequences with a DSB between the recognition site and cut site. The DNA substrate with no DSB and an MmeI recognition site is 283 bps long. For the cutting across DSB experiment, the DNA molecule with no MmeI recognition site is 164 bps long, and the DNA molecule with an MmeI recognition site is 118 bps long. The percentage cut is shown below each lane. Percentages are based on the average of three runs, with a representative gel shown. (b) MmeI digestion of pairs of DNA substrates with 1-, 2-, or 3-base overhangs at 4 °C, 20 °C, and 37 °C. (c) Bar plot quantifying the percent digestion by MmeI shown in b. Error bars showing standard deviation calculated from three replicates (n = 3). (d) Visual representation of MmeI cutting with or without a DSB.

All statistical analyses were conducted in R. ANOVA model was used to compare the degree of digestion among the different temperatures and the different overhang length groups (Fig. 1c). Pairwise comparisons were then made using the Tukey test. P-values less than 0.05 were considered to be significant.

## Results

While developing a protocol using MmeI to generate Cas9 sgRNA libraries, we noticed an unexpected product approximately 15 bp shorter than any expected products or inputs [15]. Further analysis of the digested fragment led to the hypothesis that MmeI can cut across a double-strand break (DSB) mediated by a brief interaction between complementary 2-base overhangs on each DNA molecule. To test this hypothesis, we compared in vitro digestion on synthetic oligos with one containing an MmeI recognition site at the 5′ end and 1-, 2-, or 3-base 5′ overhangs (C/G, CC/GG, and CCG /GGC, respectively) to a DNA molecule of a similar sequence without the DSB. We ran 5-minute incubations at 20 °C (Fig. 1a). The result showed 78.5% digestion with a 2-base overhang, 96.6% with a 3-base overhang, and 90.6% with two sites on the same DNA molecule. A DNA molecule containing a 1-base overhang showed minimal digestion, and blunt-ended oligos with no complementary overhang produced no digestion at all (data not shown). This result demonstrated that cutting across a DSB with overhangs containing at least 2 complementary bases is as efficient or even more efficient than cutting within the same oligo.

To characterize the nature of this hybridization and compare the effect of different temperatures on cleavage efficiency, we ran digestions at 4 °C, 20 °C, and 37 °C for all three different lengths of overhangs (Fig. 1b). Except for the 1 base overhang oligos, all digests had some degree of digestion, although the total amount digested depended on the conditions used. In the case of 2-base and 3-base overhangs, increasing the incubation temperature from 4 to 20 °C increased the amount of digestion. At 37 °C, that amount decreased for both overhang lengths. There was no significant difference between 2- and 3-base overhangs at 4 and 20 °C. However, at 37°, there was a significant increase in digestion for 3-base overhangs compared to 2-base overhangs (Fig. 1c).

## Discussion

Our results demonstrate a novel activity for the Type-IIS enzyme MmeI: the ability to cut when a DSB is present between the MmeI recognition site and its cut site 20 bp away (Fig. 1d). Each MmeI has five domains (4 shown). The recognition sequence is embedded between the Methyl transferase (MTase) domain and a target recognition domain (TRD). A helical spacer separates the endonuclease domain from MTase [13]. The disorganized endonuclease domain only becomes stable and cleaves 18–20 bps away when MmeI dimerizes. Once the MmeI trimerizes/tetramerizes, the endonuclease domain of the other MmeI (attached to TRD of another DNA substrate) causes cleavage, even if a DSB is present adjacent to the MmeI recognition sequence [16].

MmeI digestion appears to be at least as rapid as cutting a single DNA with no DSB when 2- or 3-base overhangs are used under the right conditions. We believe that MmeI binding and dimerization may stabilize the endonuclease domain, which allows it to work quickly when the overhang transiently basepairs with another DNA molecule. The effects of temperature on this cutting are somewhat complicated. Surprisingly, cutting at 20 °C was more efficient than at 4 or 37 °C for both 2- and 3-base overhangs, with almost all cutting lost at 37 °C for the 2-base overhangs. Thus, there seems to be an interaction between overhang length and enzymatic activity. While we cannot decisively conclude why this is, it is possible that it is related to two competing factors: enzymatic activity and stability of the transient base-pairing in the overhang. While MmeI is active at 4 °C, its activity increases with temperature. In contrast, transient overhang interactions are more stable at lower temperatures. It is possible that 20 °C represents a “Goldilocks” temperature where MmeI is highly active, but base interactions last long enough for cutting. Further studies are necessary to determine the mechanisms explaining the temperature dependence observed here.

Our findings serve as both an opportunity and a warning for future researchers working with MmeI. On the one hand, unexpected products might be produced by MmeI digestion. Thus, experiments using MmeI binding to temporary linkers must take care to avoid having compatible ends, or unwanted off-products may be produced. On the other hand, this property may have legitimate uses. For example, some protocols call for the ligation of an adapter containing an MmeI site followed by digestion and subsequent removal of the site [4, 15]. Our findings indicate that this step can be avoided as MmeI can directly cut the DNA substrate without ligation.
